# Do Not Let it Be the Last: End-of-Life Care Decisions in the Primary Care Clinic

**DOI:** 10.51894/001c.9204

**Published:** 2019-07-01

**Authors:** My-Trang T. Dang, Zohair Ahmed, Jason M. Betcher, Saloni Kadakia, Samuel J. Wisniewski, John W. Sealey

**Affiliations:** 1 Michigan State University College of Osteopathic Medicine Authority Health, Department of Internal Medicine; 2 Michigan State University, College of Osteopathic Medicine Authority Health, Department of Internal Medicine; 3 Michigan State University; 4 Michigan State University, College of Osteopathic Medicine Authority Health

**Keywords:** end-of-life care conversations, primary care, advance care planning

## Abstract

**CONTEXT:**

For many patients, end-of-life care (EOLC) wishes are unknown and are generally only brought up during healthcare crises. During such healthcare episodes, loved ones are often distraught, and as such, can find it difficult to focus on details surrounding the event. The best place for these discussions may be in non-acute settings including primary care clinics. The purpose of this study was to examine how well a sample of patients (N = 177) in three Michigan-based primary care teaching clinics thought they and their loved ones were prepared in terms of having their EOLC wishes known.

**METHODS:**

Prospective data were collected from three Internal Medicine teaching clinics in the Metro-Detroit area through an anonymous written survey addressing EOLC issues in a 16-item cross-sectional study. Respondents were adult patients at one of three participating primary care clinics. Perceived preparedness for EOLC was measured by: 1) possibly having had a prior EOLC discussion with a healthcare provider 2) having created an Advance Directive, such as a Durable Power of Attorney (DPOA) or Living Will with medical decision preferences, 3) reported preferences for quality versus quantity of life, and 4) preferences for CPR and other specific life-sustaining interventions.

**RESULTS:**

In this sample, 77 (43.5%) of 177 respondents had discussed their EOLC wishes with a provider. Regarding Advance Directives, 63/177 (35.6%) had established a DPOA, and 59/177 (22%) had made a Living Will. The majority of respondents preferred quality over quantity of life. The most difficult EOLC questions included the decision for CPR, tracheostomy, and PEG tube placement.

**CONCLUSIONS:**

Based on these results, EOLC discussions probably occur infrequently in the primary care or other healthcare settings. Most survey responses indicated that sample patients were unprepared concerning the details of future EOLC decisions. Engagement of such discussions should be a part of routine visits in the primary care clinic and should be re-visited when there is a change in a patient’s health. Further larger-scale studies using validated surveys are required in this vitally important area of practice. Key Words: advance care planning, end-of-life care conversations, primary care

## INTRODUCTION

Although every person’s life will eventually end, we frequently fail to consider our end of life wishes until faced with a healthcare crisis. Topics of death and dying may be rarely discussed with healthcare providers and considered taboo.^1,2^ In a report by Hamel et. al, 69% of Americans stated that death is a subject people generally avoid discussing.^1^

In addition, patient’s loved ones often feel burdened making end-of-life decisions, and it can be difficult for healthcare providers to discuss end-of-life-care (EOLC) goals under rushed conditions.^3–5^ As such, the best setting for EOLC discussions may be in non-acute settings, like primary care clinics.^4,5^

Although 92% of people in the United States feel that EOLC discussions are important, it has been estimated that only about 32% have reported having had such a discussion with healthcare providers and 18-36% of adults have formal documentation regarding their EOLC preferences.^6–8^ Documentation of these wishes are a part of an advance directive. Advance directives help specify the type of treatment a person may or may not want in the event that they are unable to express their EOLC wishes.

Advance directives may include a Durable Power of Attorney (DPOA) for Healthcare, a Living Will, a Do-Not-Resuscitate (DNR) Declaration, and a Declaration for Anatomical Gift.^9^ A DPOA, which generally specifies a Healthcare Proxy or Patient Advocate, is an individual who you select to speak on your behalf and make decisions regarding your medical treatment when you cannot make your own decisions. These documents vary from state to state are designed to help ensure that one’s end-of-life preferences are observed. Currently, in the authors’ state of Michigan, there are no standard forms and different varieties exist online.^9^

Advance care planning (ACP) is a process where individuals discuss their EOLC wishes with their loved ones and healthcare providers to direct future care.^8–10^ There is a frequent misconception that ACP should only be initiated when one becomes old or ill.^6–11^ However, these are times when it may be too late and may occur at times of crisis. A more optimal setting for EOLC discussions may be in primary care clinics.^9,10^

### Study purpose

The purpose of this study was to examine how well a sample of patients (N = 177) in three Michigan-based primary care teaching clinics thought they and their loved ones were prepared in terms of having their EOLC wishes known.

## METHODS

After IRB approval, prospective survey data were collected from September 2017 to October 2018 at three primary care Internal Medicine Clinics in the Metro-Detroit area. These three sites were teaching clinics of the resident authors. An anonymous, 16-item cross-sectional survey addressing EOLC preferences was created by the resident authors. To be eligible for the study, respondents had to have been adult patients at one of the three participating clinics.

Participation was completely voluntary, and this was emphasized to prospective respondents when the study was explained. The survey consisted of 16 dichotomous questions written at a 6-to-7^th^ grade reading level. (Table 1) An online readability checker was used to analyze the content of the survey based on a series of reading ease, grade-level and readability formulae.^12^

**Table 1.** Characteristics of study respondents (N = 177).

	**FEMALE**	**MALE**	**TOTAL**
**NUMBER OF RESPONDENTS (%)**	100 (56.5%)	77 (43.5%)	177 (100%)
**INCOMPLETE SURVEYS (%)**	31 (31%)	10 (13%)	41 (23.2%)
**MEAN AGE (SD) (YEARS)**	55.1 (16.6)	57.8 (17.3)	56.2 (16.8)

Upon completion of the survey, the resident authors asked each participant if they had any questions/concerns about the survey. If a question section was left blank, residents were encouraged to ask follow-up questions depending on the scenario and time constraints (e.g., whether patients were in a rush to leave, or other patients were waiting for the resident to see them at the clinic). Further discussions regarding their EOLC were attempted if respondents indicated, “Yes” to the last survey question, “Do you want to discuss your end-of-life care wishes with us?”

## RESULTS

### Sample demographics

A total of N = 177 adult patients across three Internal Medicine teaching clinics in the Metro-Detroit area received a study survey (Table 1). A total of 100 (56.7%) respondents were female and 77 (43.5%) were male. The mean age of respondents was 56.2 years (SD = 16.8). A subset of 41 (23.2%) surveys were incomplete (i.e., had at least one question that was left blank).

A total of 119 (67.2%) of respondents had thought about what they would want for their EOLC preferences. A subgroup of 77 (43.5%) had discussed this topic with someone else, 54 (70.0%) with loved ones and 23 (30%) with a physician. About half of the sample believed that their loved ones were generally aware of their EOLC wishes. Only 20 (11.3%) of respondents were interested in discussing their EOLC wishes with us. In this sample, 63 (35.6%) of respondents reported having created a DPOA and 39 (22.0%) had a Living Will that included what type of EOLC they would like to receive. (Table 2)

**Table 2.** Answers from the 16 survey questions

**SURVEY QUESTION**	**YES**	**NO**	**Unanswered**
**Have you thought about what you would want for end-of life care?**	119 (67.2%)	58 (32.8%)	0 (0%)
**Has anyone discussed with you your wishes for end-of-life care?**	77 (43.5%)	100 (56.5%)	0 (0%)
**If yes, was it a doctor?**	23 (30.0%)*	54 (70.0%)*	0 (0%)
**Do your loved ones know what your end-of- life care wishes are?**	90 (50.8%)	87 (49.2%)	0 (0%)
**Do you have any religious belief that can impact the type of medical intervention you can receive (for example, blood transfusions)?**	7 (4.0%)	170 (96.0%)	0 (0%)
**Would you like to be an organ donor?**	50 (28.2%)	120 (67.8%)	7 (4.0%)
**If you had to choose between living a long and uncomfortable life or a short and comfortable life, which one would you choose?**	(LONG, UNCOMFORTABLE) 27 (15.3%)	(SHORT, COMFORTABLE) 135 (76.3%)	15 (8.5%)
**If you had to choose between QUANTITY or QUALITY of life – which one would you choose?**	(QUANTITY) 16 (9.0%)	(QUALITY) 152 (85.9%)	9 (5.1%)
**Do you have a durable-power of attorney (a decision maker for you in the event that you lose your ability to make decisions)?**	63 (35.6%)	114 (64.4%)	0 (0%)
**Do you have a living will that states what type of medical care you would like in the event that you are terminally ill or unconscious?**	39 (22.0%)	138 (78.0%)	0 (0%)
**Do you want an all-natural death?**	150 (79.1%)	13 (7.3%)	14 (8.0%)
**In the event that your heart and lungs stop working, do you want everything to be done to bring you back (including CPR and placing a tube down your wind-pipe)?**	92 (52%)	59 (33.3%)	26 (14.7%)
**Would you want to live a long life even if you lose your independence (bed-bound and dependent on others to take care of you (feeding, bathing, etc.))?**	49 (27.7%)	121 (68.3%)	7 (4.0%)
**In the event that you are unable to eat on your own, would you want a feeding tube to be placed in your stomach to provide you with food and water?**	64 (36.2%)	87 (49.1%)	26 (14.7%)
**In the event that you cannot breathe on your own and need a breathing machine to help you breathe, do you want a tracheostomy?**	65 (36.7%)	86 (48.6%)	26 (14.7%)
**Do you want to discuss your end-of-life care wishes with us?**	20 (11.3%)	157 (88.7%)	0 (0%)

*Percentages were based on respondents who had a prior EOLC discussion (i.e., 77, not 177)

## Comfort/time and quality/quantity and all-natural death

One hundred fifty (79.1%) of 177 total respondents preferred an “all-natural death.” As shown in Table 3, 135 (76.3%) respondents indicated they preferred a short and comfortable remaining life. Respondent data in Table 4 highlighted that 152 (85.9%) preferred quality over quantity of remaining life.

**Table 3:** Preferences between participants who preferred a long, uncomfortable life and short, comfortable life

**ALL NATURAL DEATH**	**LONG, UNCOMFORTABLE LIFE**	**SHORT, COMFORTABLE LIFE**	**Unanswered**
**NO**	2 (7.4%)	11 (8.1%)	0 (0.0%)
**YES**	23 (85.2%)	115 (85.2%)	12 (80.0%)
**Unanswered**	2 (7.4%)	9 (6.7%)	3 (20.0%)
**TOTAL**	27	135	15

**Table 4:** Preferences for all-natural death between respondents who preferred quality and quantity of life

**ALL NATURAL DEATH**	**QUALITY OF LIFE**	**QUANTITY OF LIFE**	**Unanswered**
**NO**	10 (6.6%)	2 (12.5%)	1 (11.1%)
**YES**	131 (86.2%)	14 (87.5%)	5 (55.6%)
**Unanswered**	11 (7.2%)	0 (0.0%)	3 (33.3%)
**TOTAL**	152	16	9

## Questions left unanswered

From the 16 survey items, eight questions were left unanswered by some respondents. The majority of these missing responses concerned decisions for cardiopulmonary resuscitation (CPR) N = 26 (14.7%), percutaneous endoscopic gastrostomy (PEG) tube placement N = 26 (14.7%) and tracheostomy N = 26 (14.7%). This was followed by the item concerning a preference for living a “long, uncomfortable life” versus a “short, comfortable life” N = 15 (8.5%). (Table 5)

**Table 5:** Preferences for CPR, tracheostomy and PEG tube placement across respondents who preferred quality and quantity of life

**CPR**	**QUALITY OF LIFE**	**QUANTITY OF LIFE**	**Unanswered**
**NO**	60 (39.5%)	3 (18.8%)	4 (44.4%)
**YES**	89 (58.6%)	12 (75.0%)	4 (44.4%)
**UNSURE**	3 (1.9%)	1 (6.2%)	1 (11.1%)
**TRACHEOSTOMY**	**QUALITY OF LIFE**	**QUANTITY OF LIFE**	**Unanswered**
**NO**	77 (54.2%)	5 (31.2%)	5 (55.6%)
**YES**	58 (40.8%)	10 (62.5%)	3 (33.3%)
**UNSURE**	7 (5.0%)	1 (6.3%)	1 (11.1%)
PEG TUBE *	**QUALITY OF LIFE**	**QUANTITY OF LIFE**	**Unanswered**
**NO**	89 (58.6%)	5 (31.2%)	5 (55.6%)
**YES**	56 (36.8%)	10 (62.5%)	3 (33.3%)
**UNSURE**	7 (4.6%)	1 (6.3%)	1 (11.1%)

CPR = Cardiopulmonary resuscitation

PEG Tube = percutaneous endoscopic gastrostomy tube

## DISCUSSION

In the United States, 32% of individuals have discussed EOLC goals with someone, with 18-36% completing an advance directive.^1^ In this smaller study, our findings were similar (i.e., 35.6% had a DPOA and 22.0% had created a Living Will). One of the most frequently reported reason for not having an advanced directive may be lack of awareness in both primary care and non-primary healthcare settings.^13^ This was also reflected in our discussion with participants. In addition to the lack of awareness, many respondents in this study verbally indicated to the resident investigators that “my family will know what to do.” Research has demonstrated that deferring these difficult decisions to family members without guidance can lead to future conflicts.^10–14^

Unfortunately, individuals have been shown to have great difficulty making accurate predictions regarding their future quality of life when in compromised health states.^15–18^ Ideally, EOLC discussions should occur when patients have full decision-making capacity and quality time to fully reflect on what their preferences are.^10,14,15^

Although 119 (67.2%) respondents indicated that they had thought about their EOLC goals, only 77 (43.5%) had actually discussed it with someone. Only 20 (11.3%) participants were interested in having further discussion regarding their EOLC wishes with us in the clinic. However, for half (i.e., over 56%) of these participants, this had been their first EOLC discussion with anyone. The low percentage of interest may be related to individuals’ preference for having EOLC discussion with loved ones instead of physicians due to the sensitive nature of the topic.^6,19,20^

Unfortunately, talking about dying and death is still not a cultural norm in the United States.^1,2,21^ For some individuals, it remains a taboo topic.^1,2^ In this study, only a few respondents indicated that they were interested in having further EOLC discussion with the resident authors. Some indicated that this was likely “out of fear,” perhaps “thinking about it could be a jinx (due to superstition).” Others indicated a belief that “God will decide on what happens to me.” Although, some commented that the survey prompted them to initiate a discussion with their loved ones regarding ACP. This general hesitancy matches the findings of Fried et al. in 2009^18^ and Sudire, et al. in 2017.^22^

Another 2017 study suggested that many individuals may require a rather high level of certainty before accepting end-of-life treatment options.^23^ This high level of certainty required to make a decision to avoid feelings of regret can be very difficult to accomplish, likely contributing to the difficult nature of many EOLC decisions.^23^ In addition, Jordan et al. noted that many patients may lack an understanding of resuscitation procedures or be unable to make informed decisions before receiving structured education.^24,25^ Similarly, individuals tend to overestimate their chance of survival after inpatient CPR.^26^ Studies have demonstrated that patients’ understanding and preferences for life-prolonging interventions can be affected through the use of structured communication tools that helps to improve health literacy.^24,27^

For many individuals, having a good death entails the prevention of financial and emotional burden to their loved ones.^1,14^ Individuals with ACP are three-times as likely to have their EOLC wishes known and followed.^28^ Additionally, their loved ones experienced less anxiety, stress, and depression during bereavement.^27^ Discussion of EOLC wishes also decreases hospitalization and intensive treatments at the end of life while increasing the use of hospice care.^29,30^

When this project was initially planned, the authors were not aware of any system in place for healthcare providers to determine if their patients had an advance directive. For healthcare providers, accessibility to these documents is important. At the conclusion of this study, the authors discovered that Making Choices Michigan has recently provided access to an online database of advance directives through Great Lakes Health Connect. (See the Supplementary Materials or https://makingchoicesmichigan.org/documents/.)

As of January 2016, the Centers for Medicare and Medicaid Services (CMS) added ACP services to the Medicare Physician Fee Schedule under CPT codes 99497 and 99498. Both encounter codes are used when healthcare providers have a face-to-face discussion with Medicare patients regarding ACP.^31^ ACP is an optional component of a patient’s Annual Wellness Visit and can be incorporated during that time to avoid any patient copays. For private insurances, ACP coverage is variable.^6,31^ For further details on documentation of ACP, please see the Supplementary Materials.

### Study Limitations

The non-validated survey consisted of dichotomous “Yes/No” answers that forced participants to choose one side or the other. A third choice of unsure could have been listed on the survey for respondents to visualize as an option instead of leaving the question blank. Time constraints may have hindered eliciting fuller answers from patients and impeded their availability for further EOLC discussions with authors. Unfortunately, response rates were not measured and the demographics of individuals that declined participating were not collected. Our limited results may not reflect the patient population in other primary care clinics.

## CONCLUSIONS

Although EOLC issues remain a difficult topic to discuss, provider initiation of these discussions during primary care encounters can increase patient awareness and further engagement with loved ones or healthcare providers. Awareness of an individual’s EOLC wishes for a good death can result in cost-effective and high-quality care by decreasing hospitalization and intensive treatments at the end of life, increase hospice use, and help patients die in a manner they choose.^29,30,32^ Further studies building off the work here using validated surveys and more generalizable study samples are needed to corroborate these exploratory results.

### Conflict of Interest

The authors declare no conflict of interest.

## Supplementary Material

Supplementary Materials
