# Supplementary material for: Do Not Let it Be the Last: End-of-Life Care Decisions in the Primary Care Clinic
Source: Spartan Med Res J. 2019 Jul 1;4(1):9204. doi: 10.51894/001c.9204 (PMC7746112; doi:10.51894/001c.9204)
Supplement: Supplementary Materials [file smrj_2019_4_1_9204_22824.pdf]

DO NOT LET IT BE THE LAST

Learn more about how to make your end of life care wishes known

All resources and tools are free to access!

MIPeaceofMind
MICHIGAN'S ADVANCE DIRECTIVE REGISTRY
https://www.mipeaceofmind.org

MDHHS
Michigan Department of Health & Human Services

MAKING CHOICES MICHIGAN
Discuss. Decide. Document.

A non-profit organization whose goal is to empower individuals to "advocate for their personal health by identifying their preferences for healthcare by helping people discuss, decide, and determine their preferences."

https://makingchoicesmichigan.org/

Healthcare Providers will be able to access an Advance Directive database soon!

https://gl-hc.org/community-health-record/

GLHC
GREAT LAKES HEALTH CONNECT

acp michigan

https://www.acpmich.org/

A neutral statewide network working to "provide and grow advance care planning services in their communities."

the conversation project

Institute *for* Healthcare Improvement

https://theconversationproject.org/

"A public engagement initiative with a goal to have every person's wishes for end-of-life care expressed and respected" through the Institute for Healthcare Improvement.

Medicare Reimbursement for Advance Care Planning Services

99497
1.5 RVUs

For the initial 30 minutes of ACP
(must be $\geq$ 16 minutes)

99498
1.4 RVUs

For each additional 30 minutes of ACP
(list in addition to 99497)

ACP includes discussion of end of life care wishes and Advance Directives.
Completion of an Advance Directive is not required.

In-person conversation between the patient and/or family by a Physician, NP, or PA.

No costs to patient if completed during an Annual Wellness Visit (at other times, may have a copay through Medicare Part B).

DOCUMENTATION REQUIREMENTS

Total time in minutes
Patient/loved one was "given the opportunity to decline"
Details of conversation

Adapted from:
https://theconversationproject.org/wp-content/uploads/2016/06/CMS-Payment-One-Pager.pdf
https://www.cms.gov/Outreach-and-Education/Medicare-Learning-Network-MLN/MLNProducts/Downloads/AdvanceCarePlanning.pdf
